# Veterinary Department of the University of Pennsylvania

**Published:** 1896-07

**Authors:** 


### AMONG THE COLLEGES.

### VETERINARY DEPARTMENT OF THE UNIVERSITY OF PENNSYLVANIA.

THE twenty-fourth commencement of the Veterinary Department was held on Thursday, June 11th, at 12 noon, in the Academy of Music, Philadelphia. The large hall was filled with the friends of the graduates of the several departments. Beale's Naval Battalion Band discoursed enlivening music throughout the exercises, and added much to the enjoyment of the occasion. The provost, trustees, faculties, invited guests, and graduating classes, attired in the adopted caps and gowns, marched upon the stage promptly at 12 noon. Prayer was offered, invoking a blessing upon the university, its staff of officers, graduates and students, in impressive language, by the Rev. H. C. McCook, D.D.

Some sixteen graduates received the degree of V.M.D., among them a graduate of the Veterinary Department of Ames, Iowa, and a practising veterinarian of some years' standing.

The following is the list of graduates:

Frederick deM. Bertram, Herman A. Christmann, Gilbert G. Drummond, Charles M. Frantz, Walter W. Gardiner, Charles M. Heberton, Levi Johnson, Leroy M. Land, V.S., Richard R. Mahaffy, Henry D. Martien, Charles H. Miller, John R. Mohler, A.B., F. Sidney Roop, D.V.M., Daniel G. Shumway, Arthur H. Streeter, A.B.

Dr. John R. Mohler, A.B., of the graduating class, received the J. B. Lippincott prize of one hundred dollars, having attained the highest general average in his examinations during his three years' studies.

The prize of an écraseur, offered by a friend of the department to the member of the second-year class passing the best examination in veterinary anatomy was awarded to W. J. Storm.

The exercises were concluded by a banquet at Reisser's Café in the evening, where many of the older graduates commingled with those of the class of '96, and listened to words of wisdom, wit, and history with a deal of enjoyment. Among those who responded to toasts were Dean Marshall, Profs. Adams, Conard, and Formad, City Veterinarian Hart, Dr. Conrow, and members of the several classes. Among the out-of-town alumni present were noted Drs. Lacock, of Allegheny; Conard, of West Grove; Conrow, of Burlington, N. J.; Turner, of Lahaska; Dilks, of New Jersey; Underhill, of Media; and a number of others. Profs. Pearson and Harger were much missed, both of whom were unable to participate in the festivities.

